# Tailoring Carbon Nanotubes to Enhance Their Efficiency as Electron Shuttle on the Biological Removal of Acid Orange 10 under Anaerobic Conditions

**DOI:** 10.3390/nano10122496

**Published:** 2020-12-11

**Authors:** Ana Rita Silva, O. Salomé G.P. Soares, M. Fernando R. Pereira, M. Madalena Alves, Luciana Pereira

**Affiliations:** 1 Centre of Biological Engineering, University of Minho, 4710-057 Braga, Portugal; ana.rita.silva@ceb.uminho.pt (A.R.S.); madalena.alves@deb.uminho.pt (M.M.A.); 2 Laboratory of Separation and Reaction Engineering−Laboratory of Catalysis and Materials (LSRE-LCM), Faculty of Engineering, University of Porto, 4200-465 Porto, Portugal; salome.soares@fe.up.pt (O.S.G.P.S.); fpereira@fe.up.pt (M.F.R.P.)

**Keywords:** biological reduction, acid orange 10, tailored carbon nanotubes, toxicity, by-products toxicity

## Abstract

Anaerobic treatments have been described for the biodegradation of pollutants. However, the reactions proceed slowly due to the recalcitrant nature of these compounds. Carbon nanomaterials (CNM) intermediate in, and favor, the electron transfer, accelerating the anaerobic reduction of pollutants, which act as final electron acceptors. In the present work, different carbon nanotubes (CNT) with modified surface chemistry, namely CNT oxidized with HNO_3_ (CNT_HNO_3_) and CNT doped with nitrogen in a ball milling process (CNT_N_MB) were prepared using commercial CNT as a starting material. The new CNM were tested as redox mediators (RM), 0.1 g L^−1^, in the biological reduction of the azo dye, Acid Orange 10 (AO10), with an anaerobic granular sludge, over 48 h of reaction. Methane production was also assessed to verify the microorganism’s activity and the CNM’s effect on the methanogenic activity. An improvement in the biological removal of AO10 occurred with all CNM (above 90%), when compared with the control without CNM (only 32.4 ± 0.3%). The best results were obtained with CNT_N_MB, which achieved 98.2 ± 0.1% biological AO10 removal, and an 11-fold reduction rate increase. In order to confer magnetic properties to the CNM, tailored CNT were impregnated with 2% of iron-samples: CNT@2%Fe, CNT@2%Fe_N_MB, and CNT@2%Fe_HNO_3_. The better performance of the CNT doped with nitrogen was confirmed with CNT@2%Fe_N_MB, and the magnetic character facilitated its recovery after treatment, and did not affect its good catalytic properties. No dye removal was observed in the abiotic assays, so the removal was not due to adsorption on the CNM. Furthermore, the microorganism’s viability was maintained during the assay and methane production was not affected by the presence of the CNM. Despite the toxic character of the aromatic amines formed, detoxification was observed after the biological process with thermally treated CNT.

## 1. Introduction

The growth in industrialization, associated with ineffective wastewater treatments, leads to the contamination of water resources with different pollutants, including azo dyes. Anaerobic bioprocesses have been reported as promising for the removal of these recalcitrant compounds. However, the low transformation rates of many recalcitrant compounds in the anaerobic bioprocesses, mainly due to electron transfer limitations, may represent a drawback to their application [1]. The application of redox mediators (RM) accelerates the global anaerobic reaction rates by lowering the corresponding activation energy, as they can be reversibly oxidized and reduced, acting as electron carriers in multiple redox reactions [2]. A wide range of carbon nanomaterials (CNM), such as activated carbon (AC), activated carbon fibers (ACF), graphene oxide (GO), and carbon nanotubes (CNT) [2,3,4,5,6,7,8,9], have been used as RM in chemical and biological processes for the biological reduction and detoxification of wastewaters contaminated with azo dyes and aromatic amines [8,10,11,12]. For instance, it was shown that the rates of reduction of dyes and of aromatic amines were greatly improved, in batch and in continuous bioreactors, by adding small amounts (0.1 g L^−1^) of different CNM and, in some cases, no reduction occurred in their absence [4,9,10,13]. Furthermore, these insoluble nanomaterials can be retained in bioreactors, avoiding the need for being continuously added during the process, and are easier to remove after the process [14,15,16,17].

The efficiency of CNM is related to their high specific surface area, pore size (micro, meso, or macropores), mechanical strength, the occurrence of surface functional groups, and the possibility of tailoring their surface for specific applications [18]. CNM can be physically and chemically modified to confer new functionalities and additional surface properties [9,16,19,20].

AC surfaces have been tailored by chemical oxidation treatments with nitric acid (HNO_3_), as well as thermal treatments under nitrogen atmosphere, in order to obtain materials with different surface chemical groups, which change their acidity or basicity character, without changing significantly the textural properties [9,21,22]. The prevalence of ketonic groups on the material’s surface, and the high content of delocalized π electrons on the carbon basal planes are the main factors affecting the overall kinetics of the reduction of azo dyes [9,21,23]. These electron-rich and oxygen-free sites are responsible for the high catalytic activity and basicity of CNM [9,16].

In this sense, knowledge of the surface chemistry of these nanomaterials, namely the pH_PZC_, which indicates the basicity/acidity of the CNM, and the pKa of compounds to be degraded, will facilitate the prediction of the interactions between the CNM, microorganisms, and pollutants, thus being determinants in the efficiency of the biodegradation processes [9,24,25].

In this work, CNT were tailored by oxidative (CNT_HNO_3_) and mecanothermal (CNT_N_MB) treatments, and applied to the biological removal of AO10. The mesoporous structure and high surface active sites of CNT facilitate the access and diffusion of large molecules to the CNT surface, enhancing the electron transfer for pollutants with bigger molecules, and the consequent biotransformation, when compared with other CNM, such as AC [4,7,10]. Although CNM are applied in small amounts, their recovery at the end of the processes should be facilitated, with the aim of reusing and removing them from the treated wastewater after the process, obtaining a clear effluent, and avoiding the possible spread of toxic effects into the environment. Magnetic separation is a low cost, simple, quick, and efficient method of separation. Accordingly, tailored CNT were impregnated with 2% of Fe to prepare a magnetic material. Toxicological impact assessment of the effluents after treatment is a crucial issue for the efficacy and feasibility of these biological systems. To date, there have not been any works on the application of CNT doped with N as RM. Moreover, the CNT_N used in this work were prepared by a simple method, and not using solvents, a method that is easily scalable. In addition, in this work, the toxicological evaluation of AO10, of the treated effluent, and the by-product aniline, as well as the effects of the CNM, was done by using the *Vibrio fischeri* method.

## 2. Materials and Methods

### 2.1. Chemicals

AO10 (dye content of 90%) was purchased from Sigma-Aldrich (St. Louis, MO, USA) and a stock solution of 25 mmol L^−1^ was prepared in deionized water. Aniline (purity ≥ 99.5%) was purchased from Fluka Chemie GmbH, Buchs, Switzerland, and a stock solution of 25 mmol L^−1^ was prepared in deionized water. Acetonitrile (ACN) was acquired from Fluka Chemie GmbH (Buchs, Switzerland) at the highest analytic-grade purity commercially available, and the chemicals used to prepare the macronutrients solution were purchase from Sigma-Aldrich (St. Louis, MO, USA). All chemicals were used as received and without further purification.

### 2.2. Preparation and Characterization of Carbon Materials

A commercial multi-walled CNT (NC3100TM, Nanocyl SA., Sambreville, Belgium), with 1.5 μm average length, 9.5 nm average diameter, and more than 95% carbon purity (according to the supplier’s technical data sheet) was used as the initial material. A CNT with a strong acid character and a high amount of oxygen-containing surface groups (sample CNT_HNO_3_) was prepared by oxidation of the pristine CNT with 7 M HNO_3_ in liquid phase, at boiling temperature for 3 h, according to Gonçalves et al. [5]. Briefly, the oxidation was performed using a Pyrex round bottom flask containing 300 mL HNO_3_ 7 M and 4 g of CNT, and connected to a condenser. The liquid was heated to boiling temperature with a heating mantle for 3 h. The CNT were washed with distilled water to neutral pH, dried in an oven at 110 °C for 24 h, and stored in a desiccator for later use. In addition, N-doped CNT were prepared, aiming to obtain a CNT with N-groups incorporated (sample CNT_N_MB). For that, commercial CNT were mixed with 0.26 g of N using melamine as a nitrogen precursor, and the mixture was ball milled in a closed flask without any gas flow in a Retsch MM200 equipment, for 4 h at a constant vibration frequency of 15 vibrations/s. The resulting CNT were subjected to thermal treatment under N_2_ flow (100 cm^3^ min^−1^) until 600 °C, and kept at this temperature for 1 h, as described by Soares et al. [23].

Commercial CNT were also used as support for the metal phase (Fe), in order to provide magnetic properties to the catalyst. For that propose, 2%wt Fe was supplemented to the CNT by incipient wetness impregnation from an aqueous solution of the corresponding metal salt (Fe(NO_3_)_3_). Following the impregnation step, the samples were dried at 100 °C for 24 h, treated under nitrogen flow at 400 °C for 1 h, and reduced at 400 °C in hydrogen flow for 3 h, producing the sample CNT@2%Fe [26]. The tailored nanomaterials prepared previously were also impregnated with iron, forming the samples CNT@2%Fe_N_MB and CNT@2%Fe_HNO_3_.

The textural properties of the CNM were analyzed by N_2_ adsorption isotherms at −196 °C using Quantachrome NOVA 4200e multi-station equipment, and the samples had been previously degassed in a vacuum for 3 h at 150 °C. The Brunauer-Emmett-Teller (BET) specific surface area (S_BET_) was calculated from the nitrogen adsorption data in the relative pressure range of 0.05–0.3. Thermogravimetric analysis was performed in a NetzschSTA 409 PC Luxx^®^. The analyses were carried out under a helium flow, at a heating rate of 10 °C min^−1^ from 50 to 900 °C, using two isothermal steps at 900 °C: 7 min under helium flow, and 13 min under air flow.

Elemental analysis was carried out on a vario MICRO cube analyzer from Elemental GmbH, Langenselbold, Germany in CHNS mode. Each element (CHNS) was determined by combustion of the sample at 1050 °C, and calculated by the mean of three independent measurements, using a per-day calibration with a standard compound. Oxygen analysis was carried out on a rapid OXY cube analyzer from Elemental GmbH, Langenselbold, Germany. Oxygen composition was determined by pyrolysis of the sample at 1450 °C, and calculated by the mean of three independent measurements, using a per-day calibration with a standard compound.

TEM micrographs were obtained using a LEO 960E microscope at 120 kV.

The temperature programmed desorption (TPD) profiles were obtained with a fully automated AMI-300 Catalyst Characterization apparatus, Altamira Instruments, Pittsburgh, PA 15238, USA connected to a Dycor Dymaxion Mass Spectrometer, Ametek Process Instruments, Delaware, USA. The sample (0.100 g) was placed in a U-shaped quartz tube located inside an electrical furnace, and heated up to 1100 °C at 5 °C min^−1^ using a constant flow rate of helium of 25 cm^3^ min^−1^. For quantification of the CO and CO_2_ released during the TPD experiment, calibration of these gases was carried out at the end of each analysis.

The pH at the point of zero charge (pH_PZC_) was determined by mixing 0.05 g of each carbon material with 25 mL of NaCl solution (0.01 M). The pH was adjusted with HCl or NaOH solutions (0.01 M), to obtained values between 2 and 12. The final pH was measured after 24 h under stirring at room temperature. The pH_PZC_ value of each carbon sample was determined when the curve pH_final_ vs. pH_initial_ crossed the line pH_final_ = pH_initial_.

### 2.3. Dye Biodegradation Assays

The pristine and modified CNT were tested as RM for azo dye reduction, using AO10 as a model compound. Biological reduction of AO10 was conducted in 70 mL serum bottles, sealed with a butyl rubber stopper, containing 25 mL of buffered medium at a pH of 7 with NaHCO_3_ (2.5 g L^−1^). Basal nutrients were: NH_4_Cl (2.8 g L^−1^), CaCl_2_ (0.06 g L^−1^), KH_2_PO_4_ (2.5 g L^−1^), and MgSO_4_.7H_2_O (1.0 g L^−1^). As the primary electron donor substrate, a volatile fatty acids (VFA) mixture, containing acetate, propionate, and butyrate in a chemical oxygen demand (COD) based ratio of 1:10:10, was added to the medium. Granular sludge (GS), collected from the anaerobic internal circulation reactor of a brewery wastewater treatment plant, was the inoculum, at a concentration of 2 g L^−1^ of volatile solids (VS). CNM were present at a concentration of 0.1 g L^−1^, which was chosen based on previous studies [4,26]. The medium was flushed with N_2_/CO_2_ (80%/20%) and incubated overnight at 37 °C in a rotary shaker at 105 rpm, in order to promote the consumption of the residual substrate. After the pre-incubation period, the batch bioreactors were flushed again with N_2_/CO_2_ (80%/20%), and AO10 and VFA were added from the stock solution to the desired concentration: 0.5 mmol L^−1^ and 2 g L^−1^ of COD, respectively.

Controls were also included: blank assays without substrate in the presence and absence of CNM, biological assays without CNM, and abiotic assays in the presence of CNM. All experiments were conducted in triplicate.

### 2.4. Analytical Techniques

AO10 decolorization was followed by spectrophotometry, measuring the absorbance, at the dye wavelength of maximum absorbance, 480 nm, in a 96-well plate reader (Biotek^®^ Synergy HT, Gen5 Data Analysis Software). AO10 concentration was calculated considering the determined molar extinction coefficient of the dye at 480 nm (ε_480 nm_ = 22.27 mmol L^−1^ cm^−1^). Samples were withdrawn overtime during 48 h, centrifuged, and diluted up to an absorbance of less than 1, with a fresh solution of ascorbic acid (200 mg L^−1^) to prevent the oxidation of the formed aromatic amines. First-order reduction rate constants were calculated in OriginPro software 2016, applying the following Equation (1):

*C_t_* = *C_i_* + *C*_0_*e*^−^*^t^*^/^*^k^*, (1)

where *C_t_* and *C*_0_ are the concentrations at a certain reaction time (*t*) and at initial time, respectively; *C_i_* is the offset, a value closed to the asymptotic of the Y variable (*C*) for larger time (*t*) values, and k is the first-order rate constant (d^−1^).

The reduction of the dye was also followed by high-performance liquid chromatography (HPLC) in an Ultra HPLC (Nexera XZ, Shimadzu, Japan) equipped with a diode array detector (SPD-M20A), an autosampler (SIL-30AC), degassing unit (DGU-20A5R), LC-30AD solvent delivery unit, and Labsolutions software. A RP-18 endcapped Purospher Star column (250 × 4 mm, 5 μM particle size, from MERK (Darmstadt, Germany) was used. The mobile phase was composed of the solvents: 10 mM ammonium acetate solution and ACN. The compounds were eluted at a flowrate of 0.8 mL min^−1^, at 30 °C, with the following gradient: increase from 0% to 95% of ACN over 25 min, followed by an isocratic gradient for 10 min. Prior to HPLC analysis, samples were centrifuged (10 min at 10,000 rpm) and filtered (Whatman SPARTAN syringe filters, regenerated cellulose, 0.2 μm pore size). A calibration curve was made for AO10 (0.5–0.0156 mM) and for Aniline (0.5–0.0156 mM). The rate of aniline formation was also calculated in the OriginPro software 2016, applying Equation (2):

*C_t_* = *C*_0_ + *kt*, (2)

where *C_t_* and *C*_0_ are the concentrations at a certain reaction time (*t*) and at initial time, respectively, and *k* is the zero-order rate constant (d^−1^).

VFA consumption was analyzed after 29 h of treatment, corresponding to the reaction time in which the simpler compounds were completely consumed. The analysis was performed by HPLC (Equipment Jasco, Tokyo, Japan), using a Rezex ROA-Organic Acid H+ (8%), LC Column (300 × 7.8 mm), maintained at 60 °C, and the elution flow rate was 0.6 mL min^−1^, with UV detection at 210 nm. The mobile phase was a solution of sulfuric acid (5 mmol L^−1^), and crotonic acid was used as internal standard.

The concentration of CH_4_ present in the biogas produced in each bottle, after 29 h of treatment, was determined by Gas chromatography (GC), using a GC-2014 gas chromatograph (Shimadzu, Kyoto, Japan) fitted with a Porapak Q 80/100 mesh, a packed stainless-steel column (2 m × 1/8 inch, 2 mm), and a flame ionization detector (FID). The column, injection port, and detector temperatures were 35, 110, and 220 °C, respectively. Nitrogen was the carrier gas at a flow rate of 30 mL min^−1^. Headspace gas was sampled by a 500 μL pressure-lock syringe (Hamilton). The values of CH_4_ production were corrected for the standard temperature and pressure conditions (STP). A standard sample composed of 40% of CH_4_ was injected first, followed by sample injection.

### 2.5. Toxicity Assessment with Vibrio Fischeri

Evaluation of the possible toxicity of all the samples collected after 48 h of incubation, was performed by the standard bioassay “Water quality–Determination of the inhibition effect of water samples on the light emission of *Vibrio fischeri* (Luminescent bacteria test)” method, using freshly prepared bacteria [27]. *V. fischeri* strain NRRL-B-11177 was purchased as freeze-dried reagent, BioFix^®^ Lumi, from Macherey-Nagel (Düren, Germany), and grown in our laboratory under aerobic conditions (Figure S1) in a growth medium for bioluminescent bacteria, as described in the international standard ISO 11348-3 (2007) [27,28].

The toxicity assay was adapted from the ISO 11348-1 and ISO 11348-3, performing the assays in a microplate reader (Biotek^®^ Cytation3, Fisher Scientific, Seoul, Korea), instead of a cuvette, and using the kinetic mode. A 96-well optical Btm Plt polymer base Blk plate, from Nalge Nunc™ International, was used, where each sample (100 μL) was mixed with the bacteria test suspension (100 μL). The toxicity evaluation was performed based on the bioluminescence changes when exposed to potential toxic substances.

The samples from the biological reduction assay were centrifuged (10 min at 10,000 rpm) and filtered (Whatman SPARTAN syringe filters, regenerated cellulose, 0.2 μm pore size) prior to the toxicity assay. The AO10 (0.25 mmol L^−1^), aniline (0.4 mmol L^−1^), ascorbic acid (200 mg L^−1^), and the anaerobic medium solutions were also tested. The initial concentration of AO10 at the beginning of the biological assay was 0.5 mmol L^−1^, however, due to the high turbidity of the samples, it was diluted 2 times to avoid interference with the luminescent signal. The samples withdrawn over the degradation time were equally diluted, initially to avoid the turbidity issues, and at more advanced degradation states, these samples were diluted in ascorbic acid to avoid the oxidation of the aromatic amines, making possible the evaluation of its toxicological effect.

In order to assess the effect of the CNM used, solutions containing 0.1 g L^−1^ of each material were prepared in the anaerobic medium and placed at 37 °C and 120 rpm, for 48 h (similarly to the anaerobic assays). After, samples were collected and centrifuged, and the toxicity of the supernatants was also evaluated.

Negative controls were prepared with the bacterial suspension and a solution of 2% NaCl, in accordance with ISO 11348-1 and ISO 11348-3. Potassium dichromate (K_2_Cr_2_O_7_) at a concentration of 105.8 mg L^−1^ was used as a positive control. The salinity of all the samples and solutions used in the experiment was adjusted to 2% NaCl. The pH of the samples was measured and adjusted to values between 6 and 9 with hydrochloric acid or sodium hydroxide. Oxygen concentration was higher than 3 mg L^−1^, as required, and turbidity was avoided by sample centrifugation, filtration, and dilution.

The effect of the CNM on the bacteria growth and luminescence emission was also evaluated by the addition of the nanomaterials to the growth medium, which was incubated at 20 ± 1 °C with orbital shaking at 180 rpm, over 92 h. Cell growth was evaluated through optical density analysis at 578 nm (OD_578_), and luminescence was measured in a microplate reader (Biotek^®^ Cytation3), using a 96-well optical-bottom polymer-based black plate (Nalge Nunc^TM^ International).

Luminescence inhibition (INH%) was calculated after 30 min [27,28,29], according to Equations (3) and (4):

(3) $$INH\;\left(\%\right)=100-\frac{IT_{t}}{KF\times IT_{0}}\times 100$$

(4) $$\mathrm{with}\;KF=\frac{IC_{t}}{IC_{0}},$$

where *IT_t_* is luminescence intensity of the sample after the contact time (30), *IT*_0_ is the luminescence intensity at the beginning of the assay (time 0), KF is the correction factor, which characterizes the natural loss of luminescence of the negative control, *IC_t_* is the luminescence intensity of the control after the contact time, and *IC*_0_ is the initial luminescence intensity of the negative control. The luminescence signal was recorded in relative light units (RLU/Sec).

## 3. Results and Discussion

### 3.1. Characterization of Carbon Nanomaterials

The surface and textural characteristics of the CNM are specified in Table 1. The commercial CNT used in this study are of a mesoporous material, and present a specific surface area (S_BET_) of 201 m^2^/g. Although the differences in the S_BET_ values between the different CNM are not higher, the functionalization of the CNT led to a slight increase of S_BET_ to 223 m^2^ g^−1^, for sample CNT_HNO_3_, and to 225 m^2^ g^−1^, for sample CNT_N_MB. These differences suggest that during the treatments some changes in the CNT structure may have occurred. In oxidized materials, this increase may be explained by the fact that the oxidative process leads to the opening up of the endcaps of the CNT, and creating sidewall openings [5]. The increase of S_BET_ observed for the N-doped sample may have been due to the reduction of the entanglement of the CNT during the ball milling, which leads to shorter CNT by breaking up the tubes, without affecting the tube diameters; additionally, the presence of N-surface groups may also have an attractive effect between the tubes, leading to a higher agglomeration of the sample [23,30].

In addition to the changes observed in the surface area, some differences were detected in the pore volumes (Vp), as determined from the N_2_ uptakes at a P/Po of 0.95. The Vp increases for all the modified CNT compared to the pristine CNT. Most of the pore volume results from the free space in the CNT bundles, suggesting that the functionalities introduced improved the accessibility to the tubes. It is worth noting that the increase of S_BET_ of the functionalized CNT may also be related to the increase of Vp.

The incorporation of 2% of iron on CNT samples did not introduce significant changes in the textural properties of the starting material, probably due to the low amount of iron impregnated.

**Figure 1:**
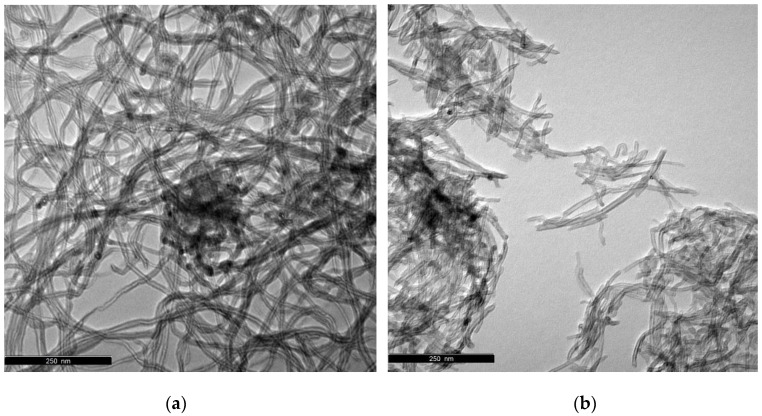
TEM images of: (**a**) CNT and (**b**) CNT_N_MB samples.

TEM micrographs of the CNT and CNT_N_MB samples shown in Figure 1 reveal that the pristine CNT were aggregates of tubes highly entangled, curved, and twisted with each other. In contrast, after the ball-milling, this high entanglement was significantly reduced, due to the mechanical treatment leading to shortened CNT, by breaking up the tubes. This observation is in line with the increase of the surface area of this sample.

TPD profiles (not shown) revealed that the pristine CNT did not present significant amounts of oxygen-containing surface groups, since the amounts of CO and CO_2_ released were very low, as can be seen in Table 2. The CNT_N_MB showed only a small increase of CO and a decrease in the CO_2_ released when compared to the original sample, indicating that the incorporation of nitrogen does not significantly increase the O-containing surface groups. These results are in line with the pH_PZC_ values obtained for these samples, which are very similar (6.6 and 6.7), revealing the neutral character of the carbon nanotubes. Contrary to this, the treatment with nitric acid strongly acidified the pristine CNT, which was due to the incorporation of a large amount of oxygen-containing groups, such as carboxylic acids, carboxylic anhydrides, lactones, phenols, and carbonyls/quinones [5,22,31], leading to a decrease of the pH_PZC_ of the original material, from 6.6 to 2.2 (sample CNT_HNO_3_), and confirming that the pH_PZC_ values are related to the surface groups in the materials, since the treatments performed on the CNM caused changes in the surface chemistry. The results of the elemental analysis showed that the original CNT were mainly composed of carbon, as expected (Table 2), with a very low percentage of hydrogen and oxygen. The low quantity of oxygen reflects the presence of low amounts, or absence, of oxygen-rich groups. CNT_HNO_3_ and CNT_N_MB are also mainly composed of carbon. The amount of nitrogen in the sample prepared by ball-milling was 1.69, showing that the incorporation of N-functionalities was successful; incorporating quaternary nitrogen (N-Q), pyrrole (N-5), and pyridinic (N-6) groups on the CNT surface [23]. The sample prepared by oxidation with HNO_3_ had a ≈ three-fold higher amount of oxygen, proving the presence of oxygen-rich groups in this CNT sample.

### 3.2. Effect of CNM on Biological Reduction of AO10

The reduction of AO10 under biologic and abiotic conditions, which caused decolorization of the medium, was followed after 48 h by spectrophotometry.

**Figure 2:**
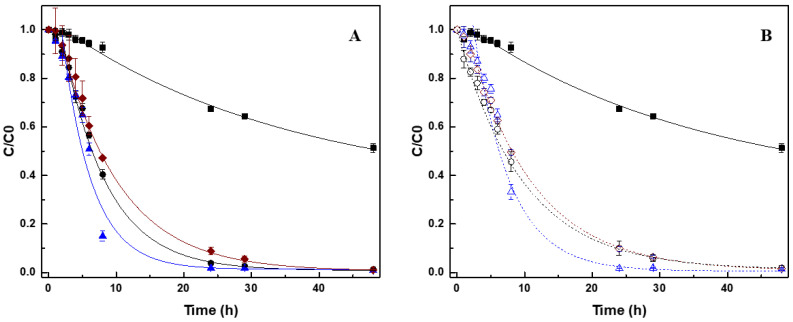
First-order rate curves of AO10 biological reduction (**A**) in the presence of different CNM: control without CNM (■); CNT (●); CNT _N_MB (▲); CNT _HNO_3_ (♦); and (**B**) in the presence of CNM impregnated with 2% Fe: control without CNM (■); CNT@2%Fe (○); CNT@2%Fe_N_MB (Δ) and CNT CNT@2%Fe _HNO_3_ (◊).

The decrease in the concentration of AO10 over time, followed first-order kinetics, as displayed in Figure 2. The removal percentage and the rate of decolorization were calculated at different conditions, and are given in Table 3. In the control assay without CNM (only GS and substrate), the biological removal of AO10 was 32.4 ± 0.3%, evidencing the recalcitrant nature of this dye. The application of all the tested CNM significantly improved the removal of the dye, and the rate of reaction. The original CNT accelerated the reduction rate 10-fold, and 97.3% decolorization was obtained. An improvement of the rate was even achieved with the treated-doped sample (CNT_N_MB), 11-times greater compared with the control without CNM, resulting in a 98.2% decolorization. On the other hand, despite the acceleration of the reduction of dye, in comparison with non-mediated reaction (nine-fold higher), oxidization of the CNT decreased their efficiency.

The better results obtained with N-doped CNT were expected, as doping CNM with heteroatoms (like N) rearranges the electrons in the carbon surface, and alters the electronic properties, enhancing their stability and catalytic performance [18]. Moreover, the nitrogen atoms provide additional electrons to the material, which can improve catalytic activity [23]. The thermal treatment of CNT_N_MB also produces a material with a low content of oxygen-containing surface groups, and a high amount of delocalized π electrons on the surface; recognized as important active sites [4,10]. The increase of S_BET_ and Vp in this material is also beneficial for its efficiency as a catalyst, due to improved access of the dye, consequently, favoring the approximation of the dye to the CNM and, thus, facilitating its reduction.

On the other hand, CNT_HNO_3_ have a high content of surface electron-withdrawing oxygenated groups, which make surface access difficult for the dye, as well as making the electron transfer from the material to the dye difficult, and making it a worse electron shuttle material [5]. Previously, other studies have reported that oxidative treatment with HNO_3_ worsens the catalytic efficiency of activated carbon (AC) for the biological (with an anaerobic consortia) and chemical (with Na_2_S) reduction of dyes and aromatic amines [9,13].

Due to their amphoteric character, CNM may have positively or negatively charged surfaces, depending on the pH of the solution, and on their pH_PZC_. The surface of materials become positively charged at pH < pH_PZC_, and negatively charged at pH > pH_PZC_ [26]. AO10 is an anionic dye, and thus negatively charged when in solution. Therefore, adsorption and electron transfer are more favorable when the surface of the CNM is positively charged in solution, while electrostatic repulsion occurs between negatively charged CNM and the anionic dye, making the adsorption and electron transfer harder. CNT_HNO_3_ were negatively charged when in the reaction medium, hampering the adsorption and, therefore, the reduction of the dye. CNT_N_MB and CNT, both presented a pH_PZC_ closer to neutrality, so their behaviors may not be so closely related to the charge.

A previous study on the effect of a commercial CNT (0.1 g L^−1^) for the biological reduction of AO10 also achieved a removal of 98 ± 2%, at the rate of 3.16 ± 0.65 d^−1^ [4]. The slightly higher rate obtained was probably due to the higher S_BET_ of that CNT, 331 m^2^ g^−1^ (about 1.6-fold higher), demonstrating the importance of surface area on catalysis.

The presence of different CNT in the abiotic assays did not cause any removal of AO10 (Figure S2), indicating that the adsorption on the materials was negligible, and also that the dye was stable in the reaction medium, and at the tested conditions. It is important to note that, although CNM are suitable adsorbents, due to the high specific surface area, the amount used in this study was very low, only 0.1 g L^−1^. The low amount of CNM required to act as RM is based on the fact that the electron exchange occurs in cycles, were the RM alter between a reduced and oxidized state during the process of electron transfer.

After biological treatment it is pertinent the recover and reuse the CNM. In this sense, the tailored CNM were impregnated with 2% of Fe to give them magnetic properties and the possibility of being recycled after treatment, and they were also tested for the biological reduction of AO10. The removals also followed a first-order kinetic, and the decrease in AO10 concentration over time is present in Figure 2B. The percentage of removal, the rate of reaction (d^−1^), as well as the extent of aniline formation are presented in Table 3. In these assays, CNM impregnated with Fe showed similar behavior compared with similar CNM, but without 2% of Fe. The reaction rate for CNT@2%Fe, CNT@2%Fe_N_MB, and CNT@2%Fe_HNO_3_, was 2.00 ± 0.18 d^−1^, 2.50 ± 0.11 d^−1^, and 1.59 ± 0.23 d^−1^, respectively.

Despite the slightly decreased reaction velocity compared to the non-magnetic CNM, better performance was again obtained with the N-doped CNT obtained by the mecanothermal process. Similarly to the oxidized non-magnetic CNT, the CNT@2%Fe_HNO_3_ exhibited the worst catalytic performance.

The worse performance of the CNT incorporated with 2% of iron, when compared to the starting CNM, may be related to the slight decreasing of the surface area (Table 1), which hinders the access and adsorption of large molecules, such as AO10, and reflected in lower reaction rates [4,13].

Despite this, an AO10 removals of 94.1 ± 1.4%, 98.1 ± 0.1%, and 93.5 ± 0.6 were obtained for CNT@2%Fe, CNT@2%Fe_N_MB, and CNT@2%Fe_HNO_3_, respectively. Therefore, likewise for modified CNT without Fe, the magnetic CNT improved the AO10 reduction comparatively to the control assay without CNM, maintaining the good catalytic properties of the tailored nanocatalysts, and also having the advantage of being easily removed from the treated water after the bioprocess by applying a magnetic field.

No removal of AO10 was obtained in the abiotic assays, contrary to previous reports in the study carried out by Pereira et al. [26], in which abiotic reduction of an azo dye in the presence of 1 g L^−1^ of CNT@2%Fe, and 96% dye removal, was observed. The reduction of the dye was attributed to the electrons generated from the oxidation of Fe^2+^ to Fe^3+^ [26,32,33]. However, in this study, only 0.1 g L^−1^ of CNM was used, so probably the final concentration of iron was maybe not sufficient to cause remarkable effects. Besides, different commercial CNT were used as matrix for the preparation of the new CNM, which, although the same reference, came from a different batch, which may have had slight differences in surface characteristics [34,35,36].

In the blank assays with CNM and GS, but without substrate, removals of around 40% where obtained with the CNM. These results suggest that the CNM can stimulate the microorganisms, and provide faster electron transfer compared with the assay without CNM, even without substrate addition, probably because of the presence of some residual substrate (which acts as an electron donor) that was not consumed during pre-incubation. In addition, in the blank assays no by-products were detected, probably due to their low concentration, and being below the detection limit of the equipment.

### 3.3. Biological Activity During AO10 Reduction

The presence of CNM did not affect the methanogenic activity, which was maintained similarly to the control. The occurrence of biological activity was confirmed by the substrate consumption analysis over time, coupled to methane (CH_4_) production (Table S1). The VFA mixture containing acetate, propionate, and butyrate at 2 g L^−1^ COD (corresponding to 1.48 mmol L^−1^ of acetate, 8.49 mmol L^−1^ of propionate, and 5.94 mmol L^−1^ of butyrate) was analyzed, and the presence of AO10 or CNM did not influence the substrate consumption, or the methane production. At the end of the 29 h of reaction, acetic and propionic acid were not detected on samples, and butyric acid was the only VFA present. These results demonstrate that the simpler substrates, such as acetate and propionate, are initially consumed by the anaerobic granular sludge, while butyrate, which is a more complex molecule, is consumed only at more advanced times of reaction, and after the consumption of the other substrates [37,38].

Regarding the control, which presented an initial concentrations of acetate, propionate, and butyrate of 2.2 ± 0.1 mmol L^−1^, 9.8 ± 0.8 mmol L^−1^, and 6.6 ± 0.4 mmol L^−1^, respectively, total consumption of acetate and propionate occurred after 29 h, and only 5.6 ± 0.1 mmol L^−1^ of butyrate was obtained. Assuming the total conversion of the substrates through the methanogenic pathway, the CH_4_ concentration should have been 21.85 mmol L^−1^, and only 13.3 ± 0.01 mmol L^−1^ were obtained, indicating that there was no total conversion of the substrates to CH_4_ in the time of the experiment.

Furthermore, during the anaerobic digestion process, a crucial interaction between bacteria and archaea communities occurs, which is known as interspecies electron transfer (IET); however, it has been reported that bacteria contribute more to the pollutant reduction than acetogenic and methanogenic archaea [39,40,41]. For the abiotic assays, as expected, there was no VFA removal (data not shown).

### 3.4. Products and Mechanism of Azo Dye Reduction

Samples of each reactor withdrawn over time were analyzed by HPLC, aiming to verify the reduction of AO10, and identify the degradation by-products formed during the treatment (Figure 2). AO10 reduction was analyzed at 480 nm and identified at the retention time (Rt) of 10.2 min. The corresponding AO10 peak decreased over the reaction time, and after 48 h of treatment, in the presence of CNM, no AO10 was detected in solution (Figure S3).

**Figure 3:**
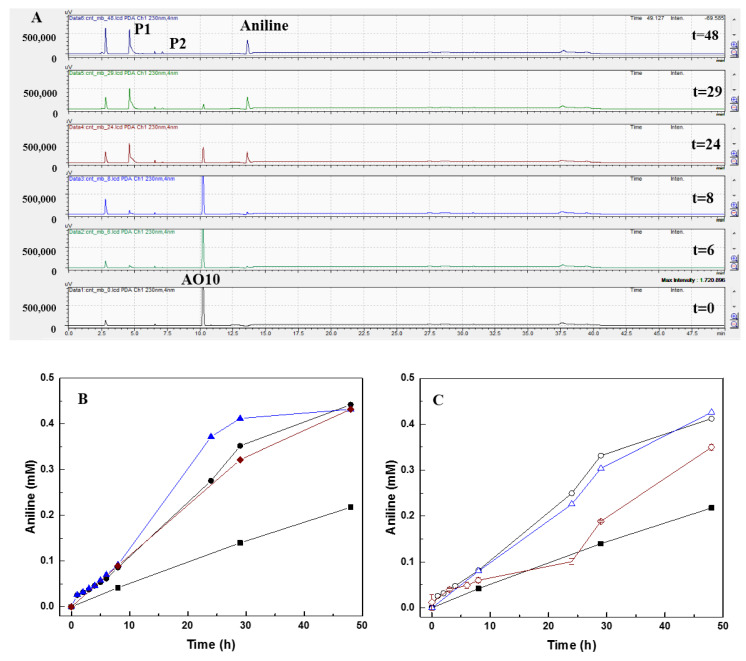
(**A**) HPLC chromatograms of the biological reduction of AO10, in the presence of CNT_N_MB, during 48 h of reaction at 230 nm. Ascorbic acid was detected at RT = 2.6 min; AO10 at RT = 10.2 min; aniline at RT = 13.6 min; undefined product P1 at RT = 4.6 min; and undefined product P2 at RT = 7.2 min. (**B**) Aniline formed during the reduction of AO10, in the presence of different CNM: control without CNM (■); CNT (●); CNT _N_MB (▲); CNT _HNO_3_ (♦); and (**C**) in the presence of CNM impregnated with 2% Fe: control without CNM (■); CNT@2%Fe (○); CNT@2%Fe_N_MB (Δ) and CNT@2%Fe _HNO_3_ (◊).

The by-products formed from the AO10 reduction were identified at 230 nm, and three new peaks were formed, P1 (Rt = 4.6), P2 (Rt = 7.2), and aniline (Rt = 13.6 min), as demonstrated in Figure 3A and Table S2. In accordance with the results obtained for AO10 reduction, formation of aniline was double in the presence of CNM (circa 0.4 mmol L^−1^) compared to the control (0.22 mmol L^−1^) (Table 3). The presence of CNM also increased the rate of product formation, and higher rates were also obtained with CNT_N_MB (Figure 3 and Table S2): 3.55-fold higher than the rate obtained in the control for aniline; 3.71-fold for P1; and 1.45-fold for P2 (Table 3). Regarding the CNT, the aniline, P1, and P2 formation increased 2.81, 1.48, and 1.45-fold, respectively, while for CNT_HNO_3_ an increase of 3.09, 2.04, and 1.44-fold, for aniline, P1, and P2, respectively, was obtained relative to the control (Table S2).

Furthermore, CNT@2%Fe_N_MB promoted an increase of 2.29-fold in aniline production, 2.03-fold for P1, and 1.81-fold for P2, demonstrating its better performance comparative to the other magnetic materials (Figure 3C and Table S2). A worse performance was observed for CNT@2%Fe_HNO_3_, and a P2 peak was not even detected (Table S2).

In biological assays, the reaction begins with the biological oxidation of the substrate (VFA), where the generated electrons are transferred through sequential reductive reactions until the final electron acceptor, in this study the azo dye, and as the reaction proceeds also the by-products. Thus, in the presence of the CNM, the electrons produced in the biological step will be accepted by the nanomaterial, which will further be transferred to the azo dye, thus reducing it. The electron shuttling efficacy of CNM, as RM, is proved by the higher reaction rates and removal extents obtained for the reduction of AO10 (Figure 2). Furthermore, the identified by-products proved that the degradation mechanism of AO10 is mediated by a reductive reaction catalyzed by the CNM [4,26], where the cleavage of the azo bond, responsible for the dye color, promoted the formation of the corresponding aromatic amine (aniline) (Figure 3) [42,43]. These results are in accordance with the work of Pereira et al. [10] and Pereira et al. [13], who similarly identified the formation of aromatic amines from the biodegradation of Acid Orange 10 and MY1, mediated by CNM. Furthermore, the presence of AC, CNT, and xerogels allowed further progress in the degradation of MY1, promoting the removal of aromatic nitroanilines, which did not happen in the absence of these CNM [13].

### 3.5. Toxicity Assessment

Evaluating the toxicity after the biological process is crucial to demonstrate if, besides degradation of the pollutant, detoxification was also achieved, or, on contrary, if there was a toxicity increase due to the by-products formed. Thus, the toxicity of the AO10 solution, and the treated solution over the 48 h of treatment, towards the biosensor *V. fischeri* was evaluated. The possible contribution of the CNM when applied in the mediated biological process was also evaluated.

The AO10 solution at the concentration of 0.25 mmol L^−1^ led to an inhibition of 35 ± 2% of the luminescence of *V. fischeri*. Regarding the biological treatment in the presence of CNM, a decrease in the toxicity of the initial AO10 solution was only obtained with CNT and CNT_N_MB: 28.4 ± 0.1% and 27 ± 2% of luminescence inhibition, respectively. For the other CNM, the final treated solution exerted higher inhibition effects (Table 4). Nevertheless, all the treated samples were considered slightly toxic, except CNT@2%Fe_HNO_3_, which was toxic (INH = 57.5 ± 9.2%) [44,45].

Over the reaction time, the toxicity extent varied according to the degradation of AO10 and the formation of by-products. For all the tested samples, the toxicity decreased during the first 5 h of biodegradation, probably due to the reduction of AO10 (Figure 2 and Figure 3), but as the reductive assay proceeded the toxicity increased again (Figure S4). In the biological process without CNM, in the first 5 h of reaction the INH dropped to 18.5 ± 6.9%, but increased again up to 23.6 ± 1.8%, after 48 h of incubation (Figure S4). Despite this increase, the value was lower than that obtained for AO10, so detoxification was obtained by the treatment (Table 4). The AO10 present in the treated solution, which was not totally removed in this condition (Figure 2), and the formed by-products (Table S2), may have both contributed to the toxicity.

A similar behavior was observed in the bioprocess catalyzed by the CNM, but after 48 h of reaction, the removal of AO10 was complete, so the inhibition of luminescence presented in those solutions was linked to the presence of the formed products, as observed by the HPLC analysis (Figure 3). Furthermore, the toxicity results obtained with CNT_N_MB are in good agreement with the degradation assay, since the best results were also obtained with this material. As the degradation of AO10 was faster with CNT_N_MB (Figure 2A), it resulted in the higher detoxification of the samples in the initial hours (INH = 14.3 ± 6.2%), and after that time an increase of luminescence inhibition up to 31.9 ± 9.0% was observed, until the 29 h of reaction, due to the cumulative toxic effect of the formed by-products, which were also formed at a higher rate, but also because of some AO10 still being present after 29 h. However, after 48 h of treatment, the toxicity decreased to 27.0 ± 2.2%, corresponding only to the toxicity of the formed products, since no AO10 was detected in the solution. On the other hand, since the rate of AO10 reduction was lower in the presence of CNT_HNO_3_ and CNT@2%Fe_HNO_3_, higher INH was observed when the treatment of AO10 was performed with oxidized CNM.

Aniline was one of the detected by-products, so its toxicity was also evaluated. At the concentration corresponding to the concentration detected for aniline at the end of the treatment, 0.4 mmol L^−1^, a luminescence inhibition of 11.7 ± 1.5% was obtained. This result demonstrates that aniline contributed to the final toxicity of the treated samples, and that other unknown by-products may also have provided an additional toxic effect. On the other hand, the toxicity of this aromatic amine at a concentration of 0.4 mmol L^−1^, was lower compared to the toxicity of the initial concentration of AO10, which means that reducing the azo dye by the anaerobic process proposed in this study, not only leads to color removal, but also to the detoxification of the dyed wastewater.

Going further in the degradation of the aromatic amines is very important to decrease the final toxicity of the treated water, and to minimize the ecological risks [46,47,48,49,50].

The presence of CNM may also have contributed to the final toxicity of the treated solution, so, the potential toxic effect of the CNM used in the biodegradation assay, was also assessed. The luminescence inhibition obtained was ≤13% for all the materials, except for CNT@2%Fe (INH = 22.3 ± 5.4%) (Table 4). Based on these results, the toxicity is considered negligible for all the tested control samples, except for CNT@2%Fe [44,45]. However, the luminescence inhibition caused by the medium itself, without CNM, was 7.5 ± 2.9%, a similar value to those obtained with the medium incubated with CNM, so this confirms that only CNT@2%Fe, at 0.1 g L^−1^, exhibited toxicity to *V. fischeri*.

The presence of CNM may have caused toxicological effects due to the release of some traces of small amorphous materials to the medium, or even due to impurities that remained in the solutions after removing the CNM. However, in this study, only a slight extent of inhibition was observed, probably due to the low amount of CNM applied. Furthermore, another study has reported that multiwalled CNT containing carboxylic and hydroxyl groups on its surface, in the concentration range of 0.5–0.875 g L^−1^, did not show any significant antimicrobial activity towards *Salmonella typhimurium*, *Staphylococcus aureus,* and *Bacillus subtilis* [51]. The introduction of oxygen-containing groups on the tips and sidewalls of the CNT during oxidation of the material, promoted a more hydrophilic surface structure and the dispersity of the CNT, avoiding the direct contact of the microorganisms with the CNT structure [7,52]. Furthermore, the oxidation of the CNT effectively removed contaminants, like trace metals, and reduced the total metal (oxide) content, which may have caused toxicity effects [7,53].

Regarding the CNM impregnated with 2% Fe, the toxicity observed was considerably higher compared to the other nanomaterials, leading to 22.3 ± 5.4% of luminescence inhibition. This higher toxicological effect that CNT@2%Fe conferred to the medium may be related to the iron that could have been released from the CNT to the medium during the incubation time. For instance, the toxicological effect of zero valent iron at nanoscale (nano-Fe^0^) was reported by several authors, since it promotes oxidative stress, damage to cell membranes, and cell death [54,55,56,57]. The toxicity mechanisms provided by nanomaterials are related to the strong affinity of iron oxides (reduced iron species, Fe^2+^ and/or Fe^0^) to the cells membrane, creating reactive oxygen species, and leading to cell death [54,55]. In addition, cell membrane disruption occurred when *Escherichia coli* was exposed to 10 mg L^−1^ of nano-Fe^0^ [55].

The toxic effect of iron was not observed in the CNT@2%Fe_N_MB and CNT@2%Fe_HNO_3_ samples, probably because the applied treatments promoted the removal of amorphous materials from CNM, and the functional groups incorporated in both treatments avoided the direct contact of the microorganisms with the sharp edges of the CNT, as mentioned above [30,58].

On the other hand, the sharp edges of the CNT may interfere with the bacteria membrane stability when CNT are in contact with the microorganism [53,59]. Taking this into consideration, the bacteria were grown in the presence of the tested CNM, but no toxic effects were observed, either in the cell density, or in the luminescent signal, relative to the control in the absence of these CNM (Figure S1). Contrarily, CNT_N_MB seemed to stimulate bacteria growth, increasing 1.1-fold the *V. fischeri* growth rate compared to the control (Figure S1A).

## 4. Conclusions

The removal of AO10 was improved by the presence of various tailored CNT, which act as RM. The surface chemistry of these materials was proved to be an important factor for the catalysis. A higher efficiency as RM was obtained by the N-doped CNT (CNT_N_MB), obtained by a mechanothermal process, reaching 98% of dye removal, and enhancing the reaction rates over 11 times, as compared with the anaerobic treatment in the absence of CNM.

Furthermore, the magnetic character of the CNM impregnated with iron allowed its easy recovery and reuse by applying a magnetic field, without the requirement of being continuously added or recovered by other additional techniques, such as filtration. Despite the advantage of these CNM being magnetic, they maintained the good catalytic properties of the tailored nanocatalysts, without changing significantly the reduction rates. In this sense, CNT_N_MB and CNT@2%Fe_N_MB were the most effective and advantageous CNM to be applied as RM. The higher efficiency for AO10 reduction and, also the detoxification obtained with those two materials, as assessed towards *V. fischeri*, makes them the best candidates to apply in anaerobic bioprocesses for anionic dyes.

Despite the detoxification achieved, the generated by-products demonstrated some toxic effects on the final treated samples, thus, raising the importance of going further in the reduction of the aromatic amines, and aiming at their total mineralization and the detoxification of the treated water. Therefore, it is imperative to accelerate the biological reactions which were demonstrated to be possible by the application of CNM.

## Figures and Tables

**Table 1:** Textural characterization of the different carbon nanomaterials (CNM).

Sample	S_BET_ (±10 m^2^/g)	Vp _p/p0 = 0.95_ (±0.005 cm^3^/g)
CNT	201	0.416
CNT_N_MB	225	0.503
CNT_HNO_3_	223	0.448
CNT@2%Fe	196	0.440
CNT@2%Fe_N_MB	243	0.581
CNT@2%Fe_HNO_3_	208	0.444

S_BET_—Specific surface area, Vp—Pore volumes.

**Table 2:** Relevant textural and chemical properties of the CNM.

Sample	(CO) _TPD_ (±20 μmol/g)	(CO_2_) _TPD_ (±20 μmol/g)	pH_PZC_	C_EA_ (wt.%)	H_EA_ (wt.%)	N_EA_ (wt.%)	S_EA_ (wt.%)	O_EA_ (wt.%)
CNT	189	81	6.6	99.8	0.11	0.00	0.00	0.06
CNT_N_MB	366	53	6.7	96.4	0.18	1.69	0.00	0.39
CNT_HNO_3_	1340	841	2.2	98.0	0.19	0.00	0.15	1.25

TPD—determined by temperature programmed desorption, EA—determined by elemental analysis.

**Table 3:** Removal (%) and rate (d^−1^) of decolorization of the AO10 solution, in the presence of 0.1 g L^−1^ of the CNM, and formation of aniline. The results include the biotic assays, the blank controls with the respective CNM and granular sludge (GS), but without substrate, and the abiotic assays with CNM in the absence of GS.

		AO10 Reduction		Aniline Formation	
Condition		Extent (%)	Rate (d^−1^)	Extent (%)	Rate (mmol d^−1^)
**Biotic assays**	**Control**	32.4 ± 0.3	0.27 ± 0.03	43.6	0.11
	**CNT**	97.3 ± 0.2	2.64 ± 0.16	88.4	0.28
	**CNT_N_MB**	98.2 ± 0.1	2.94 ± 0.18	86.4	0.35
	**CNT_HNO_3_**	94.4 ± 1.2	2.32 ± 0.14	86.4	0.27
	**CNT@2%Fe**	94.1 ± 1.4	2.00 ± 0.18	82.4	0.26
	**CNT@2%Fe_N_MB**	98.1 ± 0.1	2.50 ± 0.11	85.2	0.34
	**CNT@2%Fe_HNO_3_**	93.5 ± 0.6	1.59 ± 0.23	70.0	0.16
**Blank control**	**Control**	19.2 ± 1.1	0.09 ± 0.06	n.d.	n.d.
	**CNT**	43.3 ± 4.3	0.2 ± 0.02	n.d.	n.d.
	**CNT_N_MB**	38.7 ± 3.8	0.16 ± 0.03	n.d.	n.d.
	**CNT_HNO3**	41.5 ± 2.6	0.17 ± 0.01	n.d.	n.d.
	**CNT@2%Fe**	43.3 ± 2.9	0.18 ± 0.02	n.d.	n.d.
	**CNT@2%Fe_N_MB**	48.6 ± 1.8	0.2 ± 0.02	n.d.	n.d.
	**CNT@2%Fe_HNO_3_**	45.0 ± 3.2	0.17 ± 0.01	n.d.	n.d.
**Abiotic**	**CNT**	6.6 ± 1.3	0	n.d.	n.d.
	**CNT_N_MB**	0.6 ± 0.7	0	n.d.	n.d.
	**CNT_HNO3**	3.8 ± 1.8	0	n.d.	n.d.
	**CNT@2%Fe**	2.6 ± 1.9	0	n.d.	n.d.
	**CNT@2%Fe_N_MB**	3.5 ± 2.3	0	n.d.	n.d.
	**CNT@2%Fe_HNO_3_**	2.6 ± 1.4	0	n.d.	n.d.

n.d.—Not determined.

**Table 4:** Luminescence inhibition (INH%) of *V. fischeri* by AO10 before and after treatment (48 h), and of controls (K_2_Cr_2_O_7_, aniline, anaerobic medium, medium after incubation with 0.1 g L^−1^ of CNM), for 48 h.

Samples		INH (%)
**AO10 (0.25 mmol L^−1^)**		35 ± 2.3
**Biological degradation** **of AO10**	**Control**	23.6 ± 1.8
	**CNT**	28.4 ± 0.1
	**CNT_N_MB**	27.0 ± 2.2
	**CNT_HNO_3_**	39.7 ± 9.8
	**CNT@2%Fe**	37.9 ± 2.0
	**CNT@2%Fe_N_MB**	32.7 ± 5.6
	**CNT@2%Fe_HNO_3_**	57.5 ± 9.2
**Positive control (K_2_Cr_2_O_7_)**		86 ± 0.1
**Aniline (0.4 mmol L^−1^)**		11.7 ± 1.5
**Anaerobic medium**		7.5 ± 2.9
**Ascorbic acid (200 mg L^−1^)**		3.4 ± 0.5
**Medium after incubation with 0.1 g L^−1^ of** **CNM for 48 h**	**CNT**	6.8 ± 0.3
	**CNT_N_MB**	10.8 ± 5.3
	**CNT_HNO_3_**	7.8 ± 2.3
	**CNT@2%Fe**	22.3 ± 5.4
	**CNT@2%Fe_N_MB**	10 ± 2.1
	**CNT@2%Fe_HNO_3_**	13.0 ± 4

## Supplementary Materials

The following are available online at https://www.mdpi.com/2079-4991/10/12/2496/s1, Figure S1: Effect of CNM on *Vibrio fischeri* growth (**A**) and luminescence emission (**B**): Control without CNM (■); CNT (●); CNT _N_MB (▲); CNT _HNO_3_ (♦); CNT@2%Fe (○); CNT@2%Fe_N_MB (Δ); CNT@2%Fe _HNO_3_ (◊). Figure S2: AO10 concentration over 48 h of reaction time, in abiotic conditions, in the presence of different CNM: CNT (●); CNT _N_MB (▲); CNT _HNO_3_ (♦); CNT@2%Fe (○); CNT@2%Fe_N_MB (Δ); CNT@2%Fe _HNO_3_ (◊). Figure S3: HPLC chromatograms of biological reduction of AO10, in the presence of CNT_N_MB, during 48 h of reaction as monitored at 480 nm. AO10 was detected at RT = 10.2 min. Figure S4: Toxicity of AO10 treatment samples over the degradation time, in the presence of 0.1 g L^−1^ of CNM, towards *Vibrio fischeri*. Control without CNM (■); CNT (●); CNT _N_MB (▲); CNT _HNO_3_ (♦); CNT@2%Fe (○); CNT@2%Fe_N_MB (Δ); CNT@2%Fe _HNO_3_ (◊). Table S1: Substrate conversion, and methane production, after 29 h of biological anaerobic assays, in blank controls without dye, controls without CNM, and assays in the presence of 0.1 g L^−1^ of CNM. Table S2: Formation rate (a.u./day) and quantity (%) of each of the detected by-products of the biological reduction of AO10, after 48h of anaerobic process, in the absence (control) and presence of 0.1 g L^−1^ of different CNM.
