# Supplementary material for: Tailoring Carbon Nanotubes to Enhance Their Efficiency as Electron Shuttle on the Biological Removal of Acid Orange 10 under Anaerobic Conditions
Source: Nanomaterials (Basel). 2020 Dec 11;10(12):2496. doi: 10.3390/nano10122496 (PMC7764678; doi:10.3390/nano10122496)
Supplement: Supplementary file 1 [file nanomaterials-10-02496-s001.pdf]

nanomaterials

Supplementary Material

Table S1. Substrate conversion, and methane production, after 29 h of biological anaerobic assays, in blank controls without dye, controls without CNM, and assays in the presence of 0.1 g L^{-1} of CNM.

**Sample**	**Acetic acid (mmol L^{-1})**		**Propionic acid (mmol L^{-1})**		**Butyric acid (mmol L^{-1})**		**CH_4 (mmol L^{-1})**
**Time (h)**	**0**	**29**	**0**	**29**	**0**	**29**	**29**
**Blank**	2 ± 0.1	0	10.4 ± 0.6	0.7 ± 0.9	7.2 ± 0.8	6.9 ± 0.2	13.1 ± 0.01
**Control**	2.2 ± 0.1	0	9.8 ± 0.8	0	6.6 ± 0.4	5.6 ± 0.1	13.3 ± 0.01
**CNT**	2.3 ± 0.6	0	11.3 ± 1.1	0	7.7 ± 0.6	6.9 ± 0.5	11.9 ± 0.3
**CNT_MB**	2.7 ± 0.2	0	10.1 ± 0.5	0	6.8 ± 2.4	4.5 ± 0.8	12.4 ± 0.3
**CNT_HNO_3**	2.3 ± 0.04	0	10.6 ± 0.5	0	7.7 ± 0.1	5.5 ± 0.6	11.8 ± 0.4
**CNT@2%Fe**	3.6	0	11.7	0	8.3	5.9	13.1 ± 0.01
**CNT@2%Fe_N_MB**	3.7	0	11.7	0	8.3	7.3	12.7 ± 0.4
**CNT@2%Fe_N_HNO_3**	2.4 ± 0.1	0	10.6 ± 0.9	0	7.5 ± 01	5.7 ± 0.4	10.5 ± 0.5

Table S2. Formation rate (a.u./day) and quantity (%) of each of the detected by-products of the biological reduction of AO10, after 48 h of anaerobic process, in the absence (control) and presence of 0.1 g L^{-1} of different CNM.

	Rate (a.u./day)			Products (%)		
**Sample**	**P1**	**P2**	**Aniline**	**P1**	**P2**	**Aniline**
**Control**	2,505,302	132,558	1,271,117	65.3	3.4	31.3
**CNT**	3,705,959	192,570	3,577,374	64.3	2.5	33.2
**CNT_N_MB**	9,302,119	192,326	4,512,093	64.5	2.6	32.9
**CNT_HNO_3**	5,100,658	190,930	3,223,832	64.0	2.6	33.4
**CNT@2%Fe**	4,901,623	189,295	3,271,333	64.2	2.7	33.1
**CNT@2%Fe_N_MB**	5,076,623	239,571	2,919,967	62.9	3.4	33.7
**CNT@2%Fe_HNO_3**	3,105,069	n.d.	2,012,016	70.4	n.d.	29.6

n.d. – Not determined.

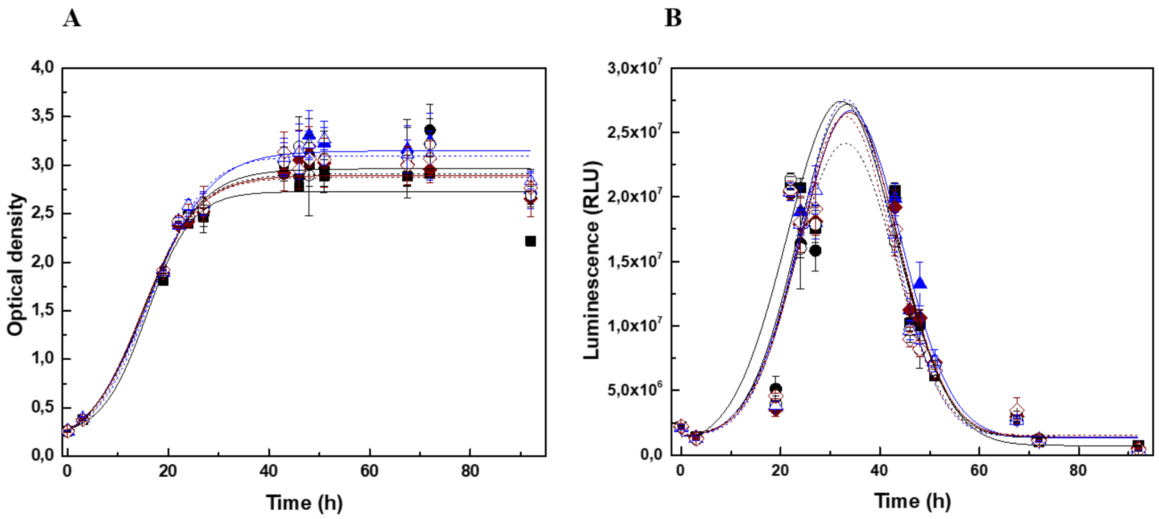

Figure S1. Effect of CNM on *Vibrio fischeri* growth (**A**) and luminescence emission (**B**): Control without CNM (■); CNT (●); CNT _N_MB (▲); CNT _HNO$_3$ (◆); CNT@2%Fe (○); CNT@2%Fe_N_MB (△); CNT@2%Fe _HNO$_3$ (◊).

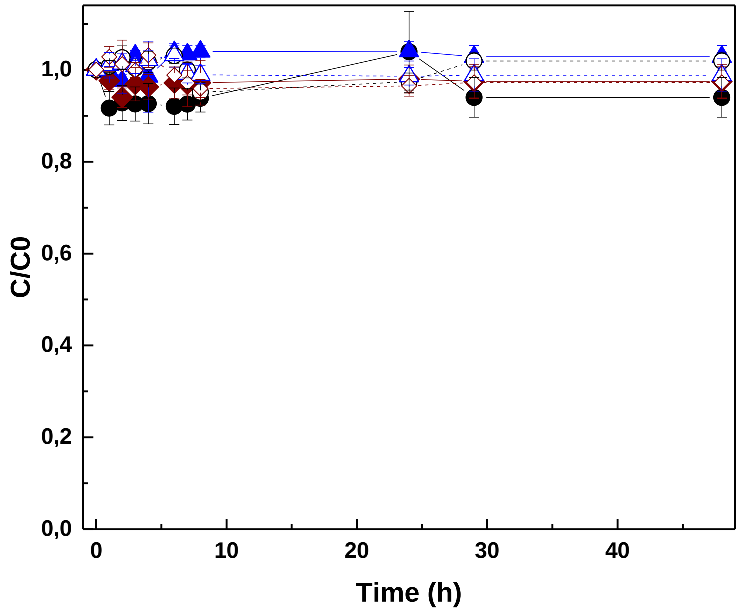

Figure S2. AO10 concentration over 48 h of reaction time, in abiotic conditions, in the presence of different CNM: CNT (●); CNT _N_MB (▲); CNT _HNO$_3$ (◆); CNT@2%Fe (○); CNT@2%Fe_N_MB (△); CNT@2%Fe _HNO$_3$ (◊).

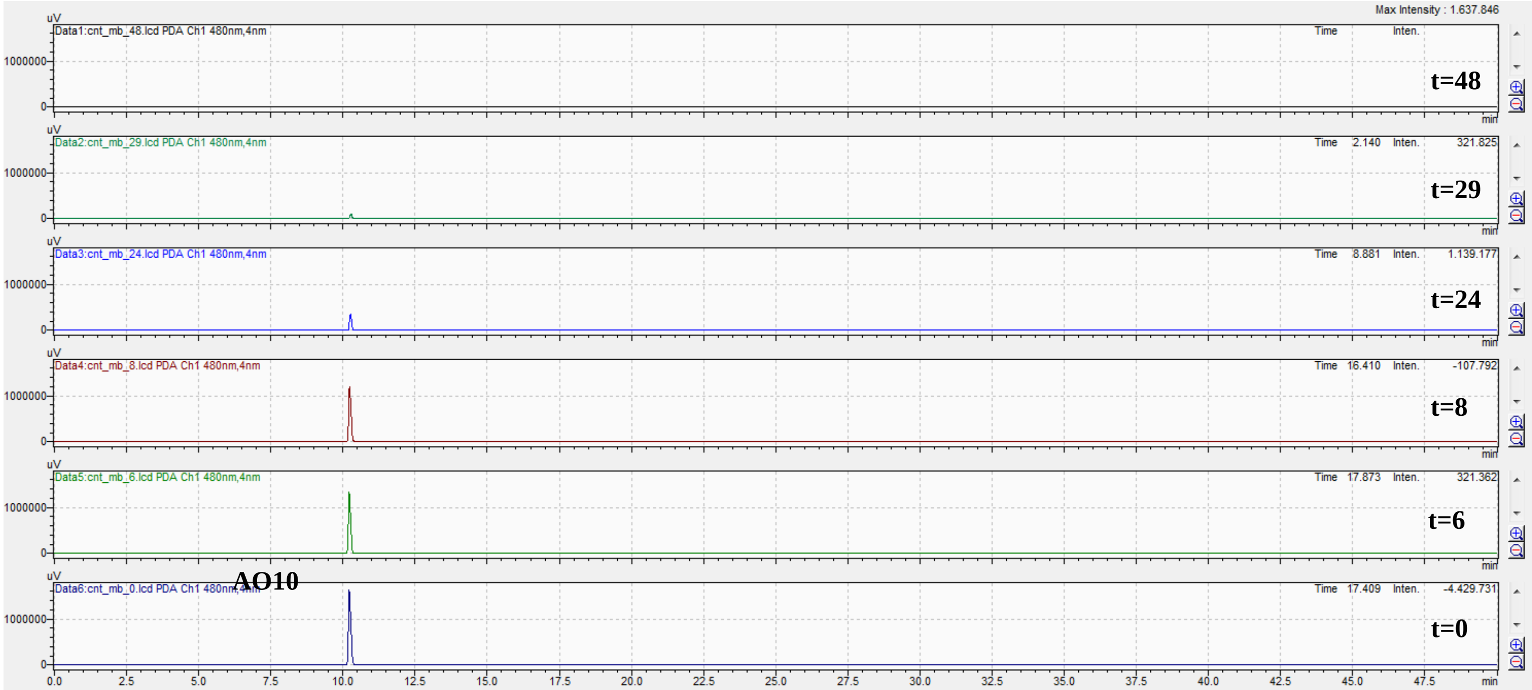

Figure S3. HPLC chromatograms of biological reduction of AO10, in the presence of CNT_N_MB, during 48 h of reaction as monitored at 480 nm. AO10 was detected at RT = 10.2 min.

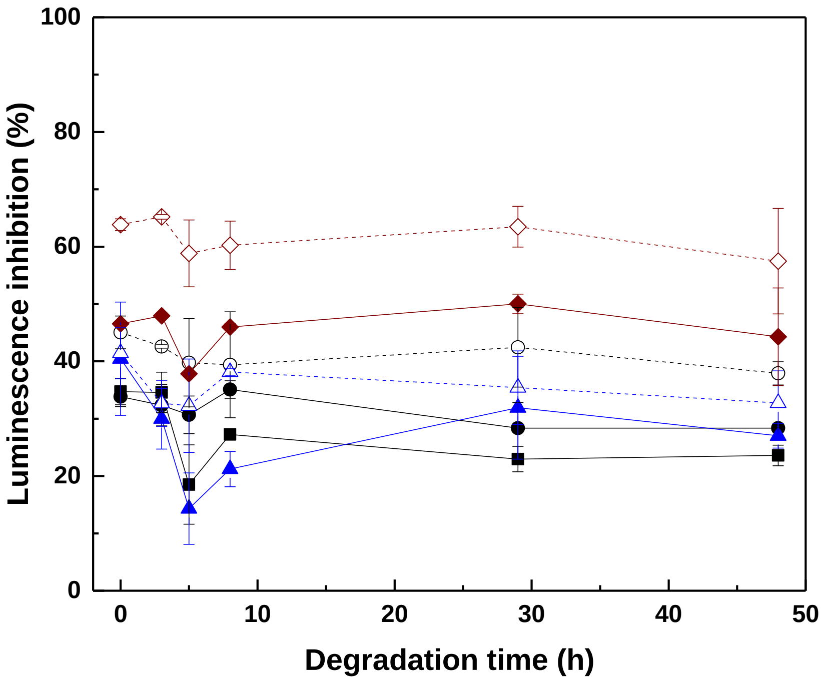

Figure S4. Toxicity of AO10 treatment samples over the degradation time, in the presence of 0.1 g L^{-1} of CNM, towards *Vibrio fischeri*. Control without CNM (■); CNT (●); CNT _N_MB (▲); CNT _HNO₃ (◆); CNT@2%Fe (○); CNT@2%Fe_N_MB (Δ); CNT@2%Fe _HNO₃ (◊).
